# SCFAs Induce Mouse Neutrophil Chemotaxis through the GPR43 Receptor

**DOI:** 10.1371/journal.pone.0021205

**Published:** 2011-06-15

**Authors:** Marco A. R. Vinolo, G. John Ferguson, Suhasini Kulkarni, George Damoulakis, Karen Anderson, Mohammad Bohlooly-Y, Len Stephens, Phillip T. Hawkins, Rui Curi

**Affiliations:** 1 Department of Physiology and Biophysics, Institute of Biomedical Sciences, University of São Paulo, São Paulo, Brazil; 2 The Babraham Institute, Babraham Research Campus, Cambridge, United Kingdom; 3 AstraZeneca Transgenic and Comparative Genomic R&D Mölndal, Mölndal, Sweden; Chinese University of Hong Kong, Hong Kong

## Abstract

Short chain fatty acids (SCFAs) have recently attracted attention as potential mediators of the effects of gut microbiota on intestinal inflammation. Some of these effects have been suggested to occur through the direct actions of SCFAs on the GPR43 receptor in neutrophils, though the precise role of this receptor in neutrophil activation is still unclear. We show that mouse bone marrow derived neutrophils (BMNs) can chemotax effectively through polycarbonate filters towards a source of acetate, propionate or butyrate. Moreover, we show that BMNs move with good speed and directionality towards a source of propionate in an EZ-Taxiscan chamber coated with fibrinogen. These effects of SCFAs were mimicked by low concentrations of the synthetic GPR43 agonist phenylacetamide-1 and were abolished in GPR43^−/−^ BMNs. SCFAs and phenylacetamide-1 also elicited GPR43-dependent activation of PKB, p38 and ERK and these responses were sensitive to pertussis toxin, indicating a role for Gi proteins. Phenylacetamide-1 also elicited rapid and transient activation of Rac1/2 GTPases and phosphorylation of ribosomal protein S6. Genetic and pharmacological intervention identified important roles for PI3Kγ, Rac2, p38 and ERK, but not mTOR, in GPR43-dependent chemotaxis. These results identify GPR43 as a *bona fide* chemotactic receptor for neutrophils *in vitro* and start to define important elements in its signal transduction pathways.

## Introduction

The short chain fatty acids (SCFAs) acetate, propionate and butyrate are by-products of anaerobic bacterial fermentation. High concentrations of these fatty acids are produced by commensal microflora in the gastrointestinal tract (concentrations ranging from 20–140 mM) and also by pathogenic bacteria at sites of anaerobic infection e.g. periodontal disease [1], [2]. There has been increasing interest in the idea that the SCFAs may play an important role in the body's immune response to the bacteria that generate them and, in particular, that they may represent an important link between diet, gut microflora and the body's inflammatory response. Further, the recent discovery that the GPR43 receptor, which is activated by SCFAs and highly expressed in neutrophils [3]–[5], may be the main mechanism through which SCFAs directly regulate immune cells and that mice lacking this receptor respond differently in mouse models of intestinal inflammation has suggested that this system may provide a novel avenue for anti-inflammatory therapy, particularly in the treatment of inflammatory bowel disease, ulcerative colitis and Crohn's disease [6], [7].

There has been little clarity, however, as to the precise nature of the effects mediated by SCFAs and the identity of the cells upon which they act. This has prevented an understanding of the effects of these molecules on the body's inflammatory response. Further, in very similar mouse models of Dextran Sulfate Sodium (DSS)-induced colitis, loss of GPR43 is reported to either ameliorate or exacerbate granulocyte infiltration and inflammation in the colon [6], [7]. One of the major obstacles to unraveling the function of SCFAs and the GPR43 receptor *in vivo* is the sheer complexity of the cells and signaling mechanisms at play in defining the delicate balance between immune system tolerance and inflammation in response to a breach in barrier integrity. There is now a substantial body of evidence which suggests that neutrophil recruitment to the intestinal mucosa is a critical player in defining levels of inflammation in this organ [8] and SCFAs have been shown to elicit neutrophil migration in Boyden chambers *in vitro* and in sterile air-pouch models *in* vivo [4], [9]. However, this effect of SCFAs is still relatively poorly characterized and has usually involved preparations of neutrophils which have already been exposed to inflammatory mediators (e.g. derived via inflammatory peritoneal exudates) or in assays which are unable to resolve effects on adhesion, motility or direction-sensing.

We have investigated in detail, the effects of gradients of SCFAs on the migration of wild type and GPR43^−/−^ mouse bone marrow derived neutrophils (BMNs) *in vitro*. Our results establish that activation of the neutrophil GPR43 receptor can support neutrophil motility and direction sensing which allows neutrophils to efficiently migrate towards a source of SCFAs. Further, we have implicated PI3Kγ, Rac2, ERK and p38 signaling pathways in the mechanism by which Gi-coupled GPR43 receptors regulate this chemotactic response.

## Materials and Methods

### Ethics Statement

All procedures were submitted to and approved by the institutional animal care committees of the Institute of Biomedical Sciences (protocol 051/livro 2) and the Babraham Institute (PPL 80/2335).

### Materials

All materials used were of the lowest endotoxin level available and were purchased from Sigma-Aldrich (Dorset, UK). ERK (PD184352) and p38 (SB203580) inhibitors were kindly provided by Simon Cook (Babraham Institute, UK). The mTOR inhibitors rapamycin and torin were purchased from LC laboratories (Woburn, USA) or kindly provided by William Festuccia (ICB -USP, Brazil), respectively. The synthetic GPR43 agonist, 2-(4-chlorophenyl)-3-methyl-*N*-(thiazol-2-yl) butanamide, also known as phenylacetamide-1, was synthesized and kindly provided by Jonathan Clark (Babraham Institute, UK).

### Mouse strains

The p110γ*^−/−^* and Rac2*^−/−^* mouse strains have been previously described [10], [11]; the GPR43*^−/−^* mouse strain was produced by Deltagen (CA, USA) [12] and provided by AstraZeneca Transgenic and Comparative Genomic R&D (Mölndal, Sweden). Strains were maintained on C57BL/6×129 backgrounds and were compared with appropriate strain-matched wild-type controls.

### Transwell assay

BMNs in a bone marrow cell suspension were assessed for their ability to migrate through polycarbonate transwell filters as described previously [13].

### EZ-Taxiscan chamber assay

Mature BMNs were purified from mouse bone marrow as previously described [14]; these preparations were 75–90% mature neutrophils based on morphology (cytospin) and FACS scan (forward/side scatter and Gr1 staining). Purified BMN were resuspended in assay buffer at 5×10^5^/ml. The EZ-Taxiscan chamber (Effector Cell Institute, Tokyo, Japan) was assembled as previously described [15]. Cell migration (at 37°C) was recorded every 60 s for 30 min using a 10× objective on Pathway 855 system (BD Biosciences). The glass coverslips used in the chamber were previously coated with 2.5 mg/ml fibrinogen.

### Analysis of migration

The *x,y* coordinates of each cell were measured using Metamorph (Molecular Devices, Sunnyvale, UK) and analysed in Mathematica (Wolfram Research, Long Hanborough, UK) using the time tracks application [16]. The speed of the cells and the migratory index of the cells (MI = distance from origin/total distance traveled) were obtained as previously described [14]. Data are expressed as the percentage of motile cells, after normalizing to account for differences in the purity of neutrophil preparations.

### Western blot analyses

Purified BMNs (1×10^6^) were stimulated with the indicated agonists at 37°C in HBSS. At the appropriate time, cells were diluted into ice-cold PBS, sedimented by centrifugation and solubilised into ice-cold lysis buffer, centrifuged (12,000 *g* for 10 min, 4°C) and the supernatants mixed with 4× SDS–PAGE sample buffer. Equal amounts of protein were subjected to SDS-PAGE and transferred to PVDF membranes. Detection was made using anti-phospho -p38 (T180/Y182), -p42/44 MAPK (T202/Y204), -PKB (S473) and –S6 ribosomal protein (S240/244) (Cell Signalling Technology, Boston, MA). Anti-β-cop (kind gift from Nicholas Ktistakis, Babraham Institute, UK) and -β-actin (Cell Signalling Technology, Boston, MA) were used as a loading control.

### Rac activation

Rac1/2 activation was assessed, using a PAK effector domain ‘pull-down’ assay, as described previously [17]. The GST-PAK-CRIB domain was purified from frozen pellets of BL21 *E. coli* cultures using glutathione sepharose™ resin. Purified BMN (2×10^6^) were stimulated with the indicated agonists at 37°C in HBSS for various times and then lysed in a 1 ml final volume of lysis buffer (20% sucrose, 10% glycerol, 50 mM Tris [pH 8.0], 0.2 mM Na_2_S_2_O_5_, 2 mM MgCl_2_, 2 mM DTT, protease inhibitors [1 mM PMSF, 1 µg/ml aprotinin, leupeptin, antipain, pepstatin A]). Lysates were centrifuged (13,000 *g*) for 3 min at 4°C to remove insolubles and then cleared lysates were incubated with 20 µl of glutathione sepharose™ 4B resin, containing approximately 20–50 µg of GST-PAK-CRIB, for 15 min at 4°C. Resin was then washed 3 times with lysis buffer and suspended in 1.66× SDS–PAGE sample buffer. An aliquot was taken from the cleared lysate and analysed for total cellular Rac. Samples were separated by 12% SDS-PAGE and analysed by Western blotting. Detection was made using anti-Rac 1 and -Rac-2 (Millipore, Bedfold, MA).

### Statistical analysis

Comparisons were performed using paired t-test and one-way ANOVA and Dunnet's multiple comparison post test using Graph Pad Prism 4.0 Software (San Diego, CA, U.S.A.). The significance was set at *p*<0.05.

## Results and Discussion

### SCFAs induce neutrophil migration through polycarbonate filters

**Figure 1:**
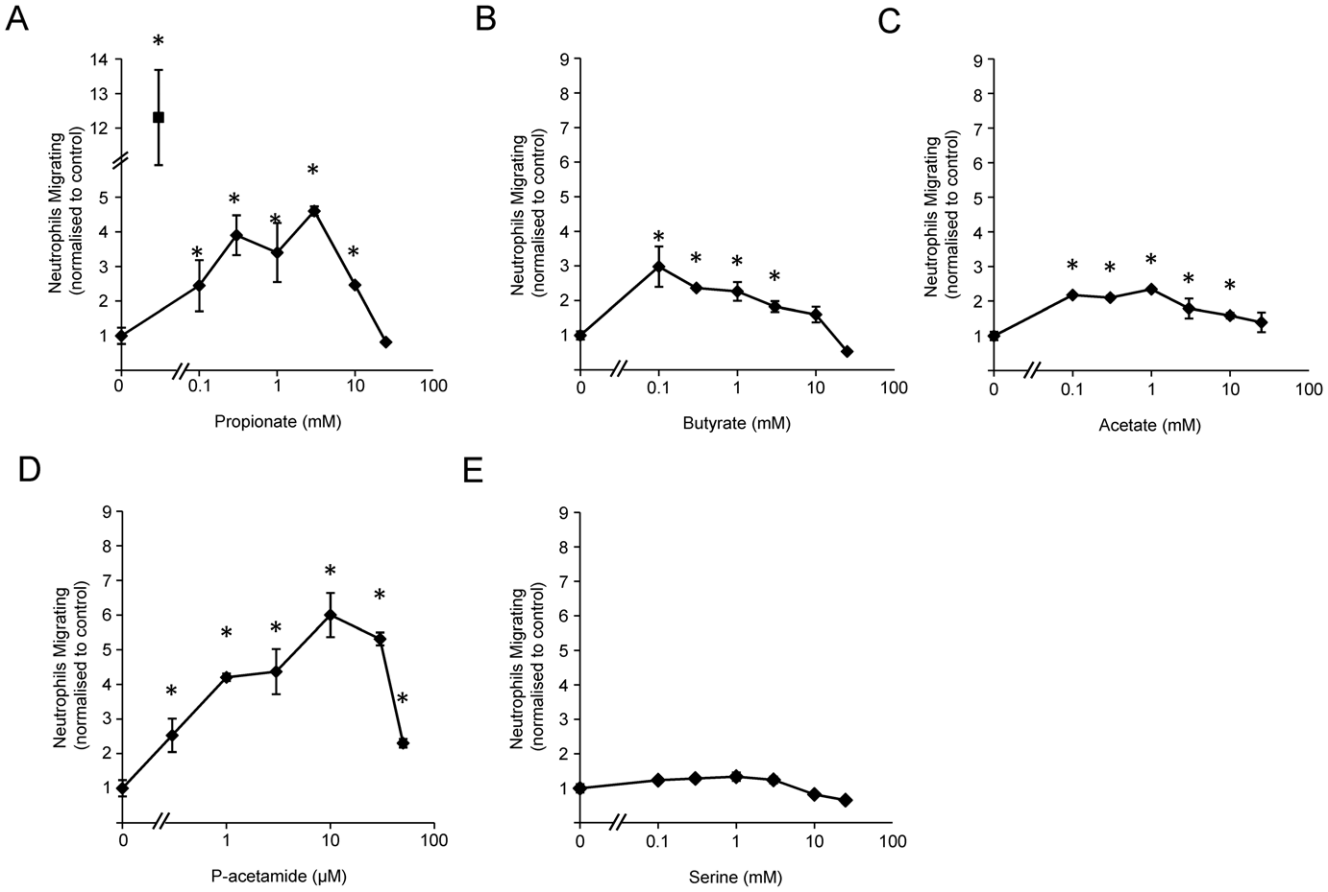
SCFAs and phenylacetamide induce neutrophil migration in a transwell chamber. Chemotaxis of BMN to the indicated concentration of propionate (**A**), butyrate (**B**), acetate (**C**), phenylacetamide (p-acetamide) (**D**) and serine (**E**) was evaluated in a transwell migration assay. The level of migration to 1 µM fMLP is shown (filled square in **A**, note scale). Data show the mean ± S.E.M. (*n* = 3) number of migrating neutrophils normalized to the control condition (non-treated). *p<0.05 compared with control condition (one-way ANOVA with Dunnet's post test).

1Initially, we tested the effect of SCFAs and a recently described synthetic agonist of the GPR43 receptor, phenylacetamide-1 [18], on the migration of BMNs through polycarbonate transwell filters. Increasing concentrations of acetate, propionate, butyrate or phenylacetamide-1 in the lower chamber significantly stimulated neutrophil transmigration with a biphasic concentration curve typical of many chemoattractants (figure 1). Optimal concentrations for migration were in the range 0.1–3 mM for acetate, propionate and butyrate and 10 µM for phenylacetamide-1, which is consistent with the previously reported effects of these agents on GPR43 receptors [7]. In contrast, serine, a structurally related substance with no activity on the GPR43 receptor was inactive in these assays (figure 1). The maximum fold increases above basal levels of migration were approximately 2.5 for acetate and butyrate, 4 for propionate and 6 for phenylacetamide-1, which are significant but relatively modest compared to an optimum concentration of the well-established chemotactic peptide fMLP (12 fold). These results extend previous work investigating the effects of SCFAs on the migration of human circulating [4] and mouse peritoneal neutrophils [6] in similar assays and suggest that these compounds may be effective chemoattractants for naïve neutrophils.

### SCFAs induce neutrophil migration in an Ez-Taxiscan chamber

**Figure 2:**
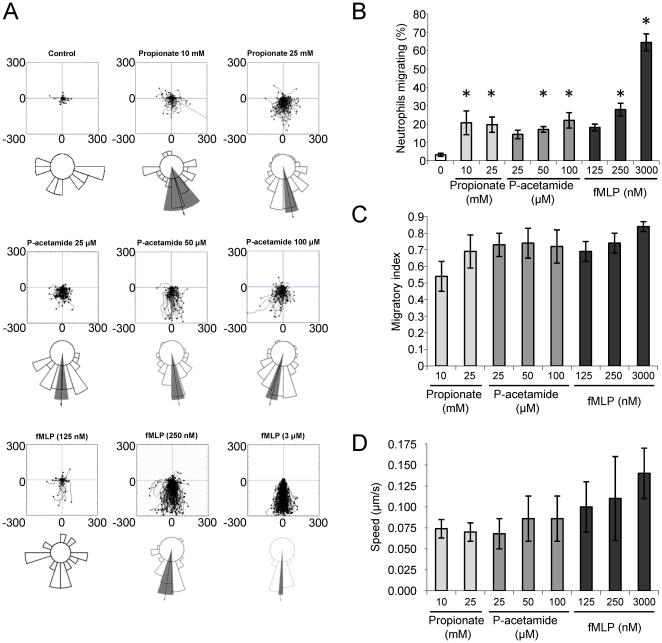
SCFAs induce neutrophil chemotaxis in the EZ-Taxiscan Chamber. Centre-zeroed tracks of purified BMN in an EZ-Taxiscan Chamber migrating towards the indicated concentrations of propionate, phenylacetamide (p-acetamide) or fMLP (the source is at the bottom of the diagram, scale is in µm) (**A**). The vector of each cell was measured as it crossed a horizon of 50 µm and these vectors are displayed as a horizon plot. Each 18° segment of the horizon plot indicates the proportion of cells that were moving within each vector. When the population was significantly moving in one direction (Raleigh test for unimodal clustering, p<0.05) the mean vector (arrow) and 95% confidence limit (shaded area) is shown. The tracks presented in **A** were analysed to show the percentage of motile cells (cells crossing a 50 µm horizon) (**B**), migratory index (of the motile cells) (**C**) and speed (of the motile cells) (**D**) (mean ± S.E.M.). *p<0.05 compared with control condition (one-way ANOVA with Dunnet's post test). The data in A–D are derived from 8–10 movies.

The chemotatic response of neutrophils to SCFAs was further examined using the Ez-Taxiscan chamber. This chamber permits a real time analysis of neutrophil movement in a well-defined gradient that is created in a narrow (5 µm) gap between two glass surfaces, one of which in these experiments is coated with fibrinogen. Using this technique it is possible to analyze distinct parameters of neutrophil movement such as directionality and speed and allows a more detailed analysis compared to filter based assays. The results obtained with purified BMNs indicated that these cells moved towards a source of propionate and phenylacetamide-1 with good speed and directionality (figure 2). Similar results were obtained with acetate and butyrate (data not shown). The concentrations of SCFAs required to induce significant neutrophil movement in these assays were an order of magnitude greater than for the analogous transwell assays, possibly because the different chamber geometries elicit different gradient profiles for a given concentration of chemoattractant and the transwell chamber presents a 3-dimensional surface for migration.

SCFAs and phenylacetamide-1 induced fewer neutrophils to move in these gradients compared to fMLP (approximately 22% versus 60%), and neutrophils moved more slowly (approximately 0.08 versus 0.15 µm/sec) (figures 2B and 2C). However, those neutrophils that did migrate to SCFAs and phenylacetamide-1 did so with a very similar migratory index to those moving in gradients of fMLP (figure 2D) and, importantly, the population was able to significantly move in one direction (as measured by the Raleigh test), suggesting these compounds were acting as true chemoattractants with a similar capacity to induce directional movement (figure 2A).

**Figure 3:**
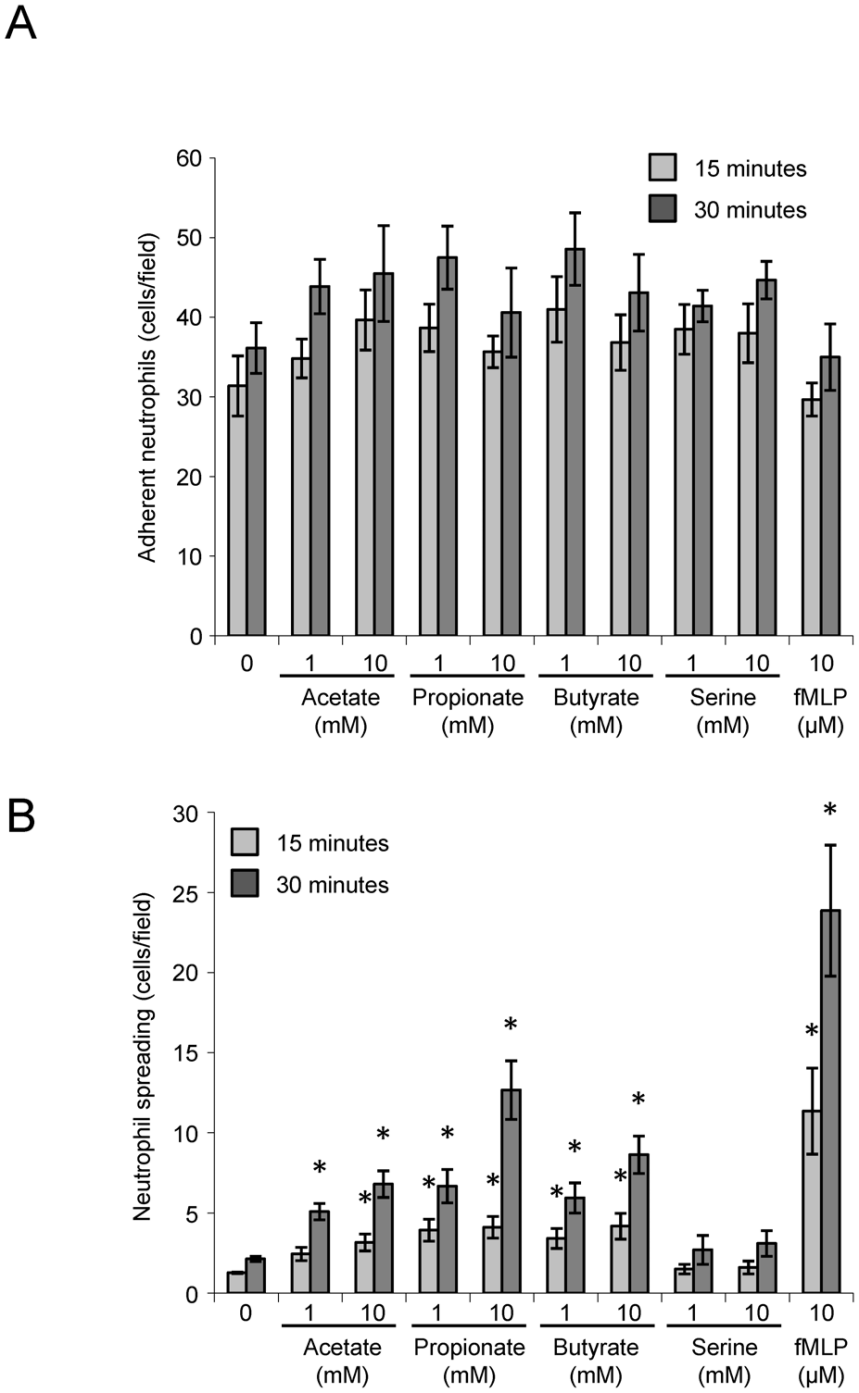
SCFAs increase neutrophil spreading on a fibrinogen matrix. Purified BMN were mixed with PBS or the indicated concentrations of agonists and immediately applied to fibrinogen coated plates for 15 or 30 min. Adherent cells were visualized by phase contrast microscopy (32×, Zeiss Axiovert) and the number of adherent (**A**) or spread (**B**) neutrophils was then quantitated (7 random fields per replicate). Data are mean ± S.E.M. (*n* = 3). *p<0.05 compared with control condition (one-way ANOVA with Dunnet's post test).

We also examined the effects of SCFAs on the ability of purified BMNs to adhere and spread on a fibrinogen matrix. Acetate, propionate and butyrate had little effect on neutrophil adhesion but all promoted a significant increase in neutrophil spreading in this assay (figure 3), suggesting this may be a component of the effects of these compounds in the Ez-Taxiscan chemotaxis assays.

### SCFAs induce neutrophil migration via the GPR43 receptor

**Figure 4:**
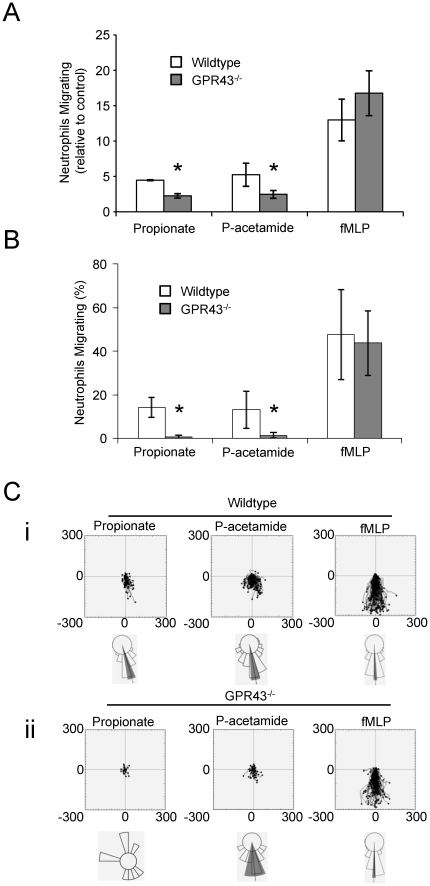
GPR43^−/−^ neutrophils do not migrate in response to propionate or phenylacetamide, but display a normal response to fMLP. Chemotaxis of wild type (WT) and GPR43^−/−^ neutrophils was evaluated in a transwell migration assay (**A**) and EZ-Taxiscan Chamber (**B–C**). Chemotaxis of wildtype or GRR43^−/−^ neutrophils in a transwell migration assay was measured to propionate (1 mM), phenylacetamide (p-acetamide, 10 µM) or fMLP (1 µM). Data show the mean ± S.E.M. (*n* = 3) number of migrating neutrophils normalized to the control condition (non-treated). *p<0.05 compared to wildtype cells (paired t-test) (**A**). Chemotaxis of wildtype or GRR43^−/−^ neutrophils in an EZ-Taxiscan chamber was measured to propionate (25 mM), phenylacetamide (p-acetamide, 50 µM) or fMLP (3 µM). The mean ± S.E.M. (*n* = 3) % of neutrophils migrating (past a 50 µm horizon) *p<0.05 compared with control condition (paired t-test) (**B**) and centre-zeroed tracks and horizon plots, as described in figure 1, (**C**) are shown. The data in B–C are derived from 6–10 movies.

The involvement of GPR43 in the chemotatic effect of SCFAs on BMNs was further investigated using neutrophils isolated from the bone marrow of GPR43^−/−^ (KO) and GPR43 wild type (WT) mice using both the transwell and Ez-Taxiscan assays. The chemotactic effect of propionate and phenylacetamide-1 on BMNs was effectively abolished in cells derived from the GPR43KO in both assay formats (figure 4). In contrast, however, neutrophils isolated from either the GPR43KO or WT mice moved equivalently in response to fMLP (figure 4). This data firmly establishes the GPR43 receptor as a selective mediator of the effects of SCFAs on the directional movement of mouse neutrophils.

These results extend previous observations suggesting the migration of mouse peritoneal neutrophils in transwell assays towards acetate [7], propionate or butyrate [6] depends on GPR43. However, the lack of effect of GPR43 on neutrophil movement in response to fMLP contrasts with previous observations that movement of GPR43^−/−^ neutrophils towards C5a or fMLP is substantially enhanced but, is consistent with the lack of effect of GPR43 on movement towards KC [6], [7]. These disparities are likely due to indirect effects stemming from the state of activation of the various neutrophil preparations used in these assays. Neutrophils characteristically undergo a process termed ‘priming’ during their preparation, caused by the presence of various pro-inflammatory substances (such as lipopolysaccharide or cytokines), that lead to increased sensitivity to subsequent activation by some stimuli [19]. The preparations of neutrophils used for both our transwell and Ez-Taxiscan chemotaxis assays were substantially unprimed, as judged by the size of the augmentation elicited by the cytokine TNFα in accepted assays of the ‘primed state’, namely Mac-1 up-regulation and fMLP-stimulated release of reactive oxygen species (figure S1).

### Propionate and phenylacetamide activate signaling pathways involving PKB, ERK1/2, p38, S6 and Rac1/2

**Figure 5:**
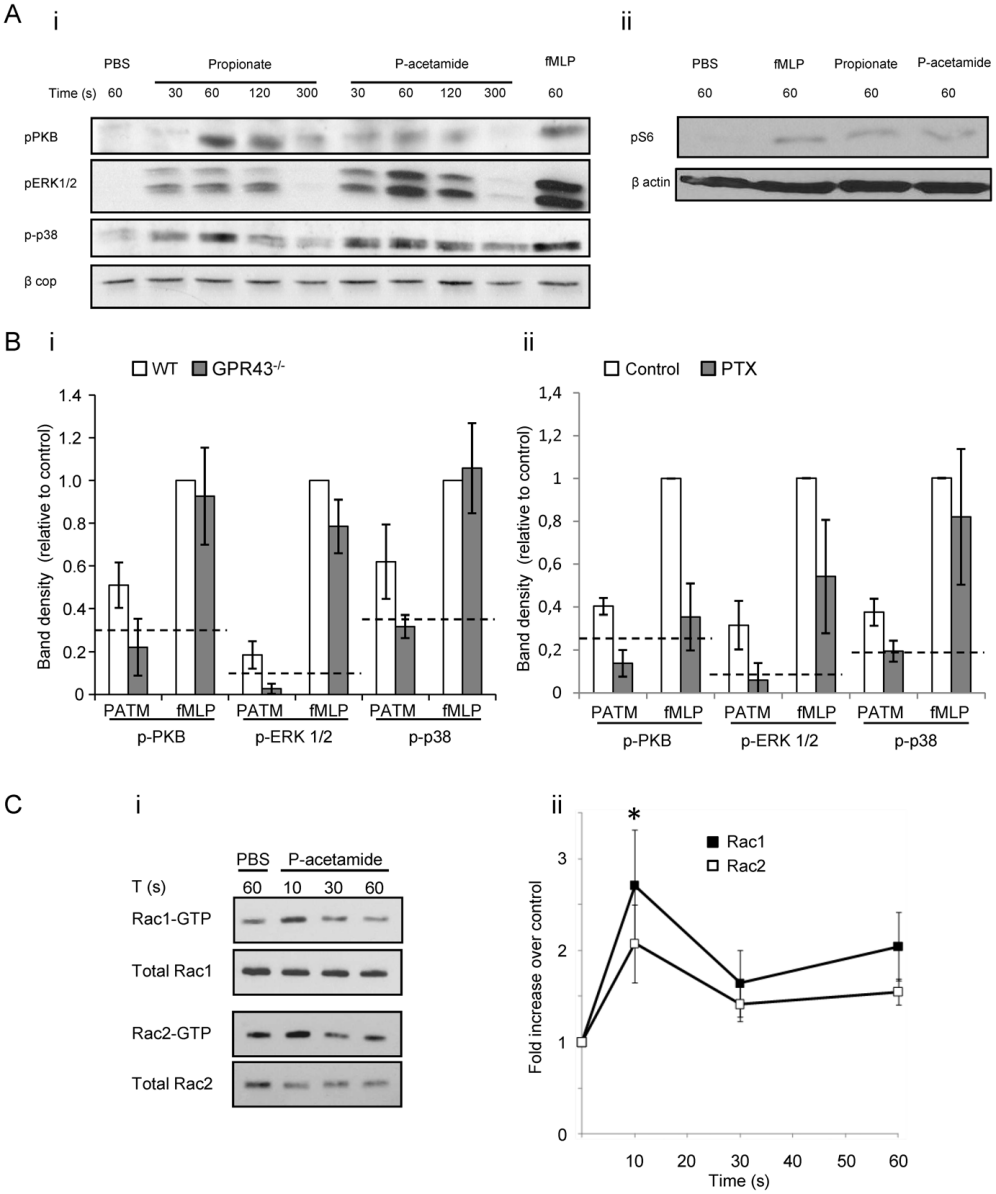
Propionate and phenylacetamide activate PKB, ERK1/2, p38 and Rac1/2 through GPR43 and Gi. Purified BMN were incubated with PBS, propionate (10 mM), phenylacetamide (25 µM) or fMLP (10 µM) for the indicated times. Whole-cell lysates were prepared and analyzed by immunoblotting using specific Abs to phosphorylated forms of PKB, ERK1/2, p38 (**Ai**) or the S6 ribosomal protein (**Aii**). The cytosolic proteins β-cop or β-actin served as loading controls. Activation of PKB, ERK1/2 and p38 by phenylacetamide (25 µM for 60 seconds; PATM) was also quantified by densitometry in neutrophils isolated from GPR43^−/−^ mice (**Bi**) or neutrophils pre-treated with pertussis toxin (PTX) (2 µg/mL) (**Bii**) and are displayed as mean ± S.E.M. (*n* = 4–5) relative to the wildtype, fMLP-treated, sample. The dashed line indicates the basal level of phosphorylation in wildtype cells (Bi) or untreated (Bii) cells. In order to assess Rac1 and Rac2 activation, purified BMN were incubated with phenylacetamide (25 µM) or PBS for the indicated time and lysates subjected to GST-PAK-CRIB pull down. A representative blot is shown (**Ci**) along with quantified data from 4 independent experiments (**Cii**) (mean ± S.E.M). *p<0.005 compared to PBS control [paired t-test]) for Rac1 and for Rac2.

Several neutrophil chemoattractants act through Gi-coupled GPCRs and phosphoinositide 3-kinase (PI3K), MAPK and Rac1/2 signaling pathways have been implicated downstream of these receptors in the regulatory networks controlling neutrophil migration [14], [20]–[23]. The GPR43 receptor has also been shown to couple to Gi in heterologous expression systems [4] and hence we initially examined the effect of both propionate and phenylacetamide-1 on the activation of the PI3K effector PKB, ERK1/2 and p38 in BMNs and the effect of genetic deletion of GPR43 or pertussis toxin (PTX)-inactivation of Gi on these responses. Both compounds induced a highly significant and time-dependent increase in phospho-PKB, ERK1/2 and p38 (figure 5Ai). Deletion of GPR43 reduced phenylacetamide-1- (figure 5Bi) and propionate-stimulated phosphorylation of PKB, ERK1/2 and p38 to basal levels but had no effect on the equivalent fMLP-stimulated responses (figure 5Bi and data not shown). Further, PTX pre-treatment (2 µg/mL during 2 h [24]) reduced both phenylacetamide-1 and fMLP-stimulated responses to a similar extent (figure 5Bii). These results strongly suggest propionate- and phenylacetamide-1-induced activation of PKB, ERK1/2 and p38 in BMNs is mediated by Gi-coupled GPR43 receptors.

Propionate and phenylacetamide-1 also stimulated the phosphorylation of the 40S ribosomal protein S6 (figure 5Aii), consistent with this being an indirect target of the PI3K/mTOR pathway [25].

We also investigated the ability of phenylacetamide-1 to activate Rac proteins in BMNs. Phenylacetamide-1 stimulated a rapid and transient increase in GTP-loading of both Rac1 (2 fold above basal) and Rac2 (3 fold above basal) (figure 5C), which is very characteristic of Gi-coupled GPCRs in this system [17].

### SCFAs- and phenylacetamide-stimulated chemotaxis requires PI3Kγ, Rac2 and MAPK pathways, but not mTOR

**Figure 6:**
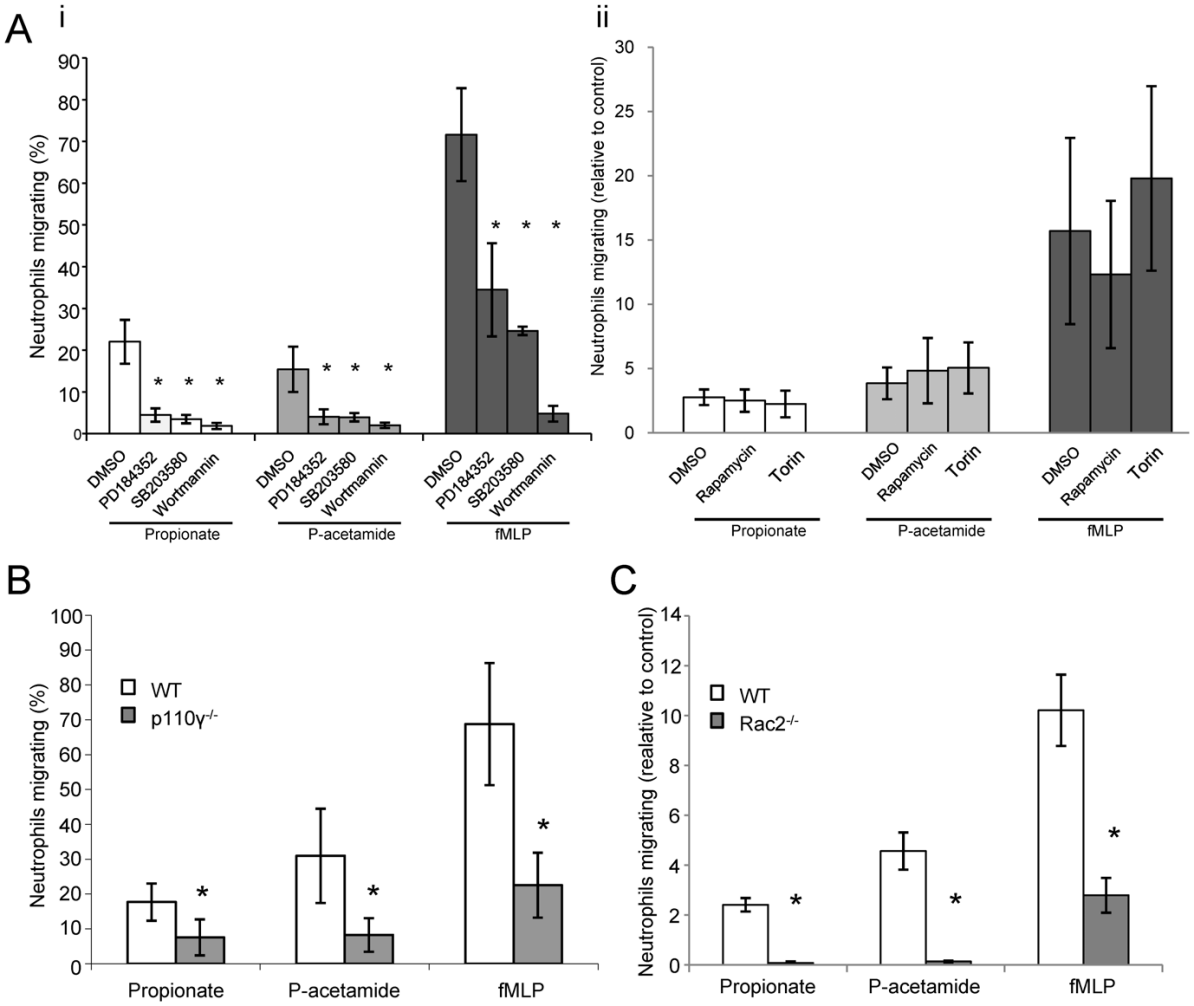
Neutrophil chemotaxis in response to propionate, phenylacetamide or fMLP requires PI3Kγ, MAPK, Rac2 but not mTor. Chemotaxis of wildtype BMN pre-incubated with the indicated inhibitor (200 nM wortmaninn, 1 µM PD184352 or 10 µM SB203580) (**Ai**), or derived from wildtype (WT) or p110γ^−/−^ mice (**B**) was assessed in response to 25 mM propionate, 50 µM phenylacetamide (P-acetamide) or 3 µM fMLP in an Ez-Taxiscan chamber. Data are expressed as the mean ± S.E.M. (*n* = 3) % of neutrophils migrating. *p<0.05 compared with control condition (cells pretreated with DMSO) (one-way ANOVA with Dunnet's post test) (**Ai**), *p<0.05 compared to the wildtype (paired t-test) (**B**). In addition, chemotaxis of wildtype BMN pre-incubated with the indicated inhibitor (100 nM rapamycin or 100 nM torin) (**Aii**), or derived from wildtype (WT) or Rac2^−/−^ neutrophils (**C**) towards 1 mM propionate, 10 µM phenylacetamide or 1 µM fMLP was assessed in a transwell chemotaxis assay and is expressed as the mean ± S.E.M. of migrating neutrophils normalized to the control condition (non-treated). The data show a representative result of 2 experiments each performed in triplicate. *p<0.05 compared to the wildtype (paired t-test).

We next evaluated the involvement of PI3K, Rac and MAPK pathways in the chemotactic response of neutrophils to propionate and phenylacetamide-1. Neutrophils were pre-incubated with 200 nM wortmaninn (inhibitor of PI3Ks), 1 µM PD184352 (inhibitor of ERK) or 10 µM SB203580 (inhibitor of p38) and then evaluated for their chemotatic responses to propionate, phenylacetamide-1 or fMLP. Inhibition of ERK, p38 and PI3K lead to a significant reduction in the number of cells migrating in response to propionate (reductions of 79%, 83% and 92%, respectively), phenylacetamide (reductions of 75%, 74% and 88%, respectively) and fMLP (reduction of 52%, 64% and 92%, respectively) (figure 6A), indicating that the activation of these pathways is important for the chemotactic effect of SCFAs on neutrophils. In contrast, inhibition of the mTORC1 complex by rapamycin, or both the mTORC1 and mTORC2 complexes by the mTOR inhibitor torin, had no significant effect on fMLP, propionate or phenylacetamide-1 stimulated chemotaxis (figure 6Aii), despite both having their anticipated effects on the mTORC1 substrate S6 and the mTORC2 substrate S473-PKB [25] (figure S1).

We also investigated the involvement of PI3Ks in these chemotactic responses further by assessing the effects of SCFAs on BMNs purified from PI3Kγ^−/−^ mice. The Class I PI3K isoform PI3Kγ has previously been implicated in neutrophil chemotaxis responses to fMLP using assays analogous to those performed here using the Ez-Taxiscan chamber [14]. We found that loss of PI3Kγ dramatically reduced the number of motile neutrophils in response to propionate, phenylacetamide-1 and fMLP (figure 6B). We also investigated chemotactic responses to phenylacetamide-1, propionate and fMLP in BMNs purified from Rac2^−/−^ mice. Rac2 has previously been implicated as a central player in the regulation of neutrophil chemotaxis downstream of Gi-coupled GPCRs and PI3Kγ [17], [20]. We found that chemotaxis responses to propionate or phenylacetamide-1 were abolished in the absence of Rac2, to an even greater extent to that seen in response to fMLP (figure 6C).

### The role of SCFAs in neutrophil migration

Our observations establish that the GPR43 receptor is a neutrophil chemoattractant receptor and is required for the directional movement of mouse neutrophils towards a source of acetate, propionate or butyrate *in vitro*. Further, they strongly suggest that GPR43 mediates these effects through pertussis toxin sensitive Gi proteins and a signaling network which includes PI3Kγ, Rac2 and MAPKs.

While it is clear that SCFAs can act as chemoattractants it is unclear if or how they would function *in vivo*. The concentrations of SCFAs required to elicit neutrophil migration *in vitro* are ≥0.1 mM, which are very high compared to other established chemoattractants. They are also extremely high compared to the concentrations of ligands required to elicit physiological responses through other GPCRs, perhaps implying that they are not the endogenous ligands for GPR43. However, these concentrations of SCFAs are well within the range that may be anticipated to occur locally at sites of anaerobic bacterial infection or at lesions of the intestinal barrier [1], [26] and thus, in the absence of a more effective competitor, it must be assumed that SCFAs will indeed interact with GPR43 at these locations. It is difficult to imagine scenarios however, where SCFAs would not be present together with other more potent chemoattractants (e.g. KC, IL-8, LTB4, C5a, fMLP, MIP-2, etc) and thus it is not clear what precise role or importance SCFAs may have in neutrophil migration *in vivo*. Further, the two studies that have started to address the role of GPR43 in neutrophil migration to sites of intestinal inflammation *in vivo* present conflicting results, suggesting this receptor may elicit indirect effects on the complex series of interactions which define neutrophil recruitment, activity and survival in this location [6], [7]. In addition, SCFAs also act on other cell types (i.e. intestinal epithelial cells, lymphocytes and macrophages) possibly through a different mechanism (by inhibiting histone deacetylase activity) and modulate the production of cytokines and chemokines involved in the recruitment of leukocytes [27]–[30]. Thus there are still many outstanding issues with regard to the function of SCFAs and the GPR43 receptor in regulating neutrophil function. Nevertheless, our results start to define the parameters for their effects on neutrophil migration *in vitro* and provide further impetus to investigate this potential interaction *in vivo*, which is likely to require the development of new tools to selectively interfere with either the production or presentation of SCFAs and their selective recognition by the GPR43 receptor on neutrophils.

## Supporting Information

Figure S1 **Priming status of BMNs.** (**A**) BMN in murine bone marrow cell suspensions were selected by immunostaining against Gr-1 and flow cytometry. Gr-1-positive cells were analysed, by immunostaining, for expression of MAC-1 in the presence or absence of 20 ng/ml TNFα. Data shown are mean ± S.D for three experiments performed in duplicate. *p<0.05, paired Student's T-test (**B**). 1×10^6^ purified BMN were pre-incubated for 1 hr at 37°C in the absence (mock primed) or presence (TNFα primed) of TNFα (4.55 ng/ml). Cells were then incubated with luminol/HRP, prior to addition of fMLP (10 µM) as described in Materials and Methods S1. Total ROS responses were measured by chemiluminesence, recorded on a 96 well plate using a Berthold Microlumat Plus luminometer, as described in Materials and Methods S1. All incubations were performed in at least duplicate. Shown are accumulated light emission over 3 min (mean ± S.E.M) from three experiments, expressed as fold of integrated response in mock primed cells. *p<0.05, paired Student's T-test.
(TIF)

Figure S2 **mTOR activation.** (**A**) Purified BMN were pre-incubated with 100 nM torin (inhibitor of mTOR 1 and 2) for 30 minutes and then stimulated with PBS, fMLP (10 µM), propionate (10 mM) or phenylacetamide (25 µM) for 60 seconds. Whole-cell lysates were prepared and analyzed by immunoblotting using specific Abs to phosphorylated forms of PKB or the S6 ribosomal protein. (**B**) Activation of PKB and S6 ribosomal protein by PBS, fMLP (10 µM), propionate (10 mM) or phenylacetamide (25 µM) was also analyzed in BMN pre-incubated with the inhibitor of mTOR 1, rapamycin (100 nM), for 30 minutes. β-actin served as loading controls.
(TIF)

Materials and Methods S1 (DOCX)
