# Supplementary figures and images for: SCFAs Induce Mouse Neutrophil Chemotaxis through the GPR43 Receptor
Source: PLoS One. 2011 Jun 15;6(6):e21205. doi: 10.1371/journal.pone.0021205 (PMC3115979; doi:10.1371/journal.pone.0021205)

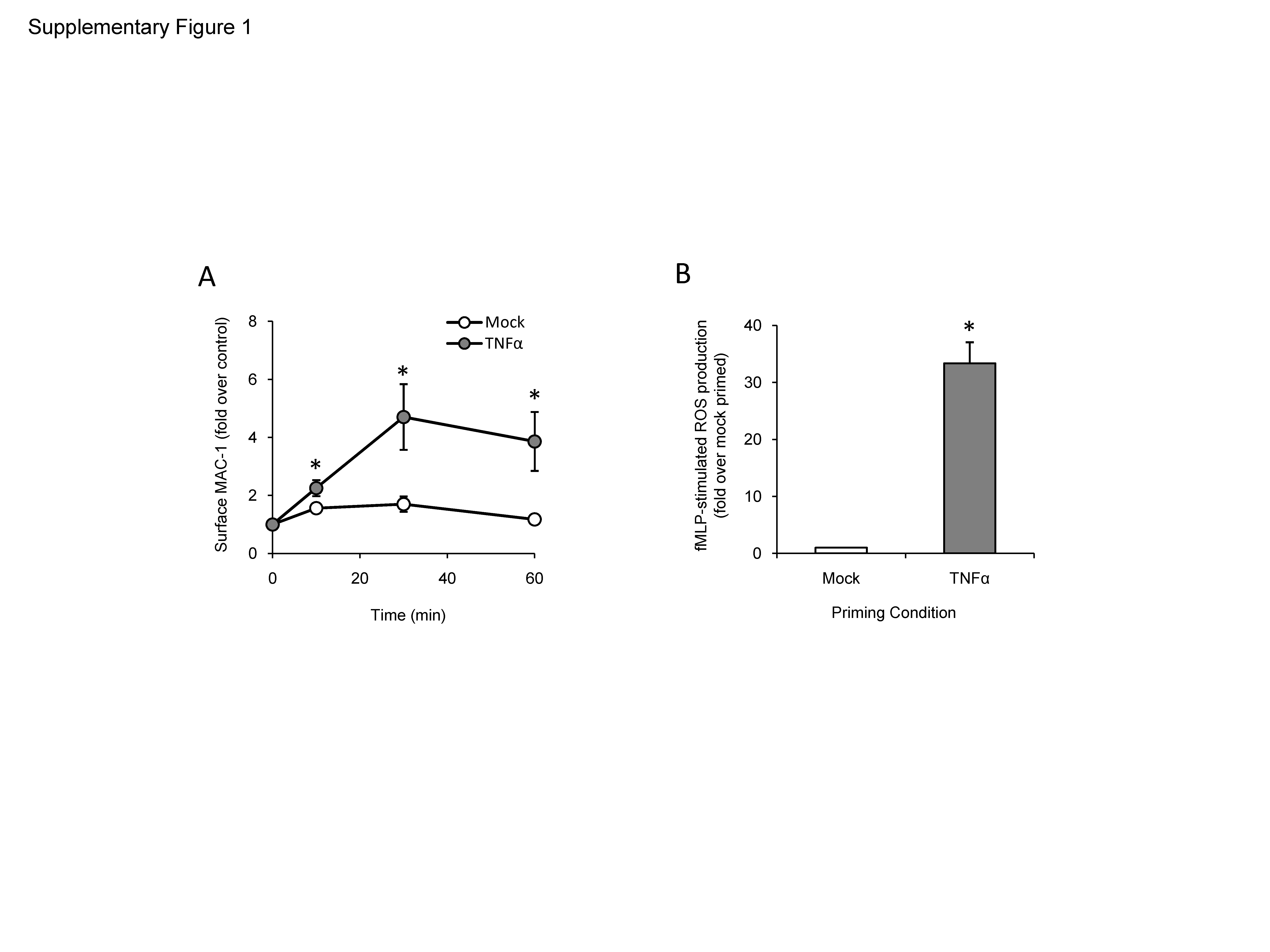

Supplement: Figure S1 — Priming status of BMNs. (A) BMN in murine bone marrow cell suspensions were selected by immunostaining against Gr-1 and flow cytometry. Gr-1-positive cells were analysed, by immunostaining, for expression of MAC-1 in the presence or absence of 20 ng/ml TNFα. Data shown are mean ± S.D for three experiments performed in duplicate. *p<0.05, paired Student's T-test (B). 1×10^6^ purified BMN were pre-incubated for 1 hr at 37°C in the absence (mock primed) or presence (TNFα primed) of TNFα (4.55 ng/ml). Cells were then incubated with luminol/HRP, prior to addition of fMLP (10 µM) as described in Materials and Methods S1. Total ROS responses were measured by chemiluminesence, recorded on a 96 well plate using a Berthold Microlumat Plus luminometer, as described in Materials and Methods S1. All incubations were performed in at least duplicate. Shown are accumulated light emission over 3 min (mean ± S.E.M) from three experiments, expressed as fold of integrated response in mock primed cells. *p<0.05, paired Student's T-test. (TIF) [file pone.0021205.s001.tif]

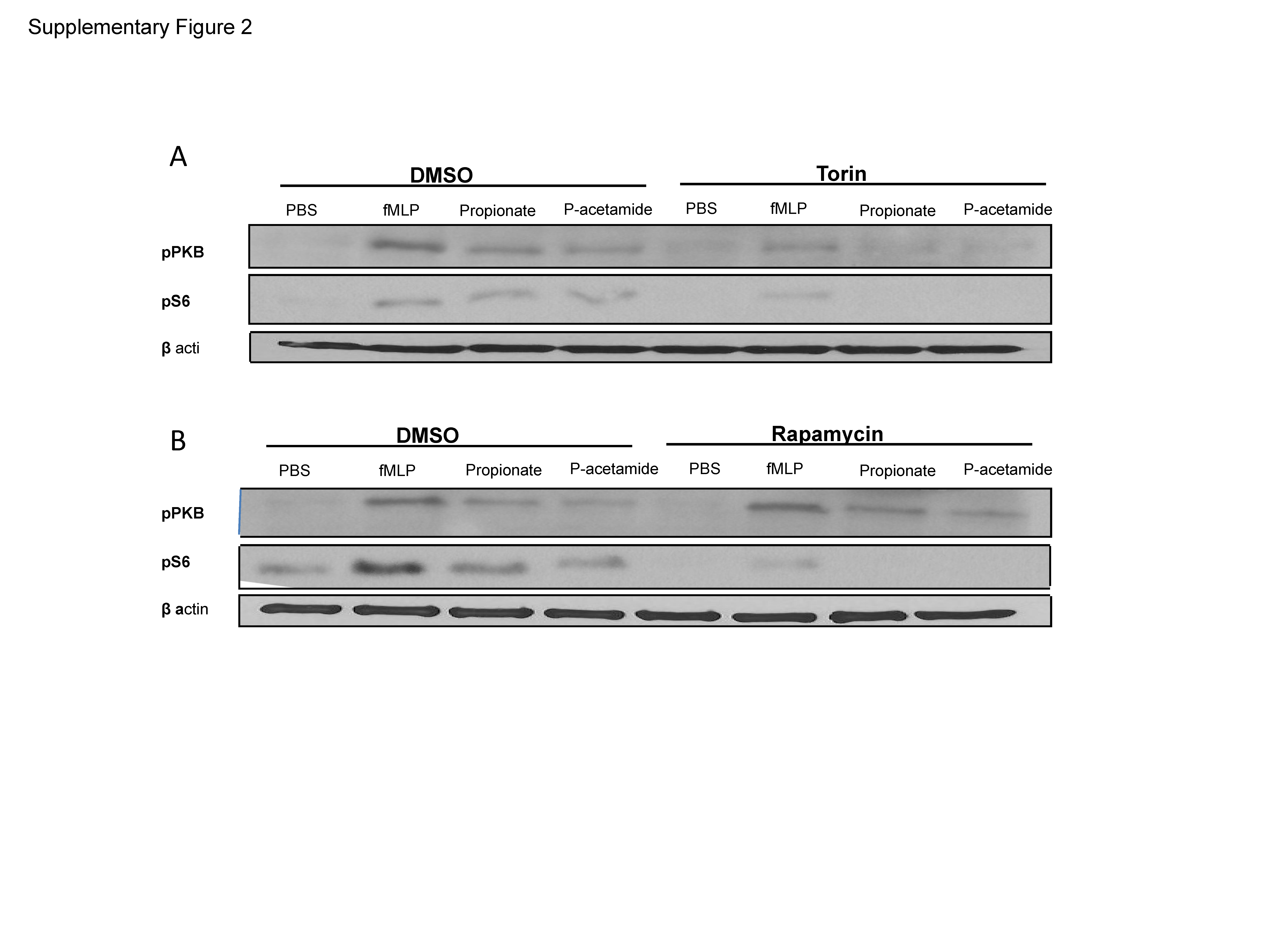

Supplement: Figure S2 — mTOR activation. (A) Purified BMN were pre-incubated with 100 nM torin (inhibitor of mTOR 1 and 2) for 30 minutes and then stimulated with PBS, fMLP (10 µM), propionate (10 mM) or phenylacetamide (25 µM) for 60 seconds. Whole-cell lysates were prepared and analyzed by immunoblotting using specific Abs to phosphorylated forms of PKB or the S6 ribosomal protein. (B) Activation of PKB and S6 ribosomal protein by PBS, fMLP (10 µM), propionate (10 mM) or phenylacetamide (25 µM) was also analyzed in BMN pre-incubated with the inhibitor of mTOR 1, rapamycin (100 nM), for 30 minutes. β-actin served as loading controls. (TIF) [file pone.0021205.s002.tif]
