# Supplementary material for: SCFAs Induce Mouse Neutrophil Chemotaxis through the GPR43 Receptor
Source: PLoS One. 2011 Jun 15;6(6):e21205. doi: 10.1371/journal.pone.0021205 (PMC3115979; doi:10.1371/journal.pone.0021205)
Supplement: Materials and Methods S1 — (DOCX) [file pone.0021205.s003.docx]

**SUPPLEMENTARY MATERIALS AND METHODS**

**Materials**

Luminol, horse radish peroxidase (HRP), and Dulbecco’s phosphate-buffered saline (PBS) with Ca^2+^ and Mg^2+^ were from Sigma-Aldrich. Murine TNFα was from R&D Systems. All buffer components were from Sigma-Aldrich and were endotoxin-free or low-endotoxin, as available.

**Flow cytometric analysis of MAC-1 expression**

Murine bone marrow cell suspension was isolated and suspended in HBSS^++^, passed through a 40μm cell strainer, centrifuged (326 *x g* for 5 min at 4^o^C) and re-suspended at 5x10^6^ cells/ml in D-PBS^++^/0.1% BSA. Cells were stimulated with an equal volume of D-PBS^++^/0.1% BSA containing 40ng/ml murine TNFα (20ng/ml final conc.) and incubated with rotation at 37^o^C. At the indicated times, the reaction was quenched by addition of 1ml ice-cold D-PBS^++^/0.1% BSA and cells were centrifuged (12,000 *x g* for 1 min at 4^o^C) and re-suspended in D-PBS^++^/0.1% BSA containing 1μg/ml Rat anti-Gr-1-APC along with 5μg/ml Rat anti-MAC-1-PE or isotype control Rat IgG_2b_-PE (eBioscience). Cells were labelled for 30 min on ice then centrifuged (12,000 *x g* for 1 min at 4^o^C) and re-suspended in D-PBS/0.1% BSA. Samples were analysed on a FACSCalibur flow cytometer and data interpreted using FlowJo (v7.4.1) software.

**Measurement of ROS Production**

Purified BMN were pre-incubated for 1hr at 37°C in the absence (mock primed) or presence (TNFα primed) of TNFα (4.55 ng/ml). Rate kinetics of total ROS production were then measured using a luminol-based assay in 96-well plates (Berthold Technologies) essentially as described previously^1^. Briefly, 5x10^5^ cells were incubated with luminol (150µM) and HRP (18.75U/ml) for 10min at 37ºC. Cells were then added manually to fMLP (10µM final), and measurement started immediately. Light emission was recorded by a Berthold Mircolumat Plus luminometer (Berthold Technologies). Data output is total relative light units per second integrated over 3min.

**Supplementary References**

1. Anderson KE, Boyle KB, Davidson K, et al. CD18-dependent activation of the neutrophil NADPH oxidase during phagocytosis of Escherichia coli or Staphylococcus aureus is regulated by class III but not class I or II PI3Ks. Blood. 2008;112:5202-5211.
